# How does the role of complementary and alternative medicine in general practice differ between countries? Interviews with doctors who have worked both in Germany and elsewhere in Europe

**DOI:** 10.1186/s12906-024-04624-w

**Published:** 2024-09-03

**Authors:** Klaus Linde, Robert Bayer, Jan Gehrmann, Bianca Jansky

**Affiliations:** 1 TUM School of Medicine and Health, Department Clinical Medicine, Technical University Munich, Institute for General Practice and Health Services Research, Munich, Germany; 2 TUM School of Medicine and Health, Department Health and Sports Sciences, Chair of Social Determinants of Health, Technical University Munich, Munich, Germany; 3 Medical Faculty, Institute for Ethics and History of Health in Society, University of Augsburg, Augsburg, Germany

**Keywords:** Complementary and alternative medicine, Qualitative study, General practice, Country comparison, Germany, Italy, Netherlands, Norway, UK

## Abstract

**Background:**

Available data suggest that general practitioners (GPs) in Germany use complementary and alternative medicine (CAM) modalities more frequently than GPs in many other countries. We investigated the country differences perceived by general practitioners who have worked in Germany and in one of four other European countries with regard to the role of complementary and alternative treatments in primary care.

**Methods:**

In this qualitative study we conducted semi-structured interviews with 12 GPs who had worked both in Germany and Italy, the Netherlands, Norway or the United Kingdom (UK; *n* = 3 for each of the four countries). Participants were asked how they perceived and experienced country differences regarding health system, relevance of CAM modalities, the role of evidence-based medicine (EBM) and science, and how they handle so-called indeterminate situations. For the analysis, we followed a thematic analysis approach according to Braun and Clarke with focus on themes that cover CAM.

**Results:**

Participants unanimously reported that they perceived CAM to be more relevant in general practice in Germany compared to the other countries. We identified four overarching themes in relation to the perceived reasons for these differences. Firstly, physicians with experiences in countries with a strong EBM and science orientation (Netherlands, Norway and the UK) considered the deeply ingrained view in national healthcare systems and GP communities that CAM modalities are not evidence-based as the main reason for the lower use of CAM by GPs. Secondly, extensive training of communication skills was cited as a reason that reduced the need for CAM in the Netherlands, Norway and the UK. Thirdly, differences in patient expectations and demands were perceived as a factor contributing to greater utilisation of CAM by German GPs compared to the other countries. Finally, country-specific reimbursement mechanisms were considered as a factor influencing the role of CAM in general practice.

**Conclusions:**

The study results point to major differences between countries with regard to the role of CAM in GP care. Differences in basic attitudes in the discipline of general practice, patient expectations and system conditions appear to play an important role here.

**Supplementary Information:**

The online version contains supplementary material available at 10.1186/s12906-024-04624-w.

## Background

The use of complementary and alternative medicine (CAM) modalities varies strongly across countries. In a nationally representative survey in 21 European countries, the use of CAM by the population was highest in Germany (40%) [1]. While there is a lack of high quality international comparative studies, the available data also suggests that the use of CAM modalities by general practitioners (GPs) is more prevalent in Germany than in many other western counties [2–8]). In a national survey, 85% of participating GPs reported prescribing or applying at least one CAM treatment once weekly or more often [7]. Herbal remedies (77%), vitamins and supplements (41%) and homeopathic preparations (32%) were the most frequently reported modalities.

CAM encompasses a wide range of very diverse diagnostic and therapeutic methods. One typical defining characteristic of a method as CAM is that it “falls outside of mainstream healthcare” [9]. German academics who advocate “science-orientated medicine” are very critical of the widespread use of CAM by German GPs and other doctors [10]. CAM modalities play only a very minor role in medical studies and specialist training. A CAM-sceptical attitude prevails at medical faculties and university institutes of general practice. Still, there is a long-standing tradition of interest in ’natural’ and non-conventional treatments both in the population as well as in the medical profession [11]. German physicians can obtain additional designations for various CAM procedures, which are recognised by the German Medical Association. In addition to doctors, around 47,000 CAM practitioners (‘Heilpraktiker’) are offering their services [12]. Social health insurance (which covers about 90% of the German population) pays only for very few CAM interventions, but the willingness to pay out of pocket is high among many patients [13].

In two earlier qualitative studies, one of the authors investigated the question why so many German GPs use CAM modalities in their medical practice and how they justify this by interviewing highly experienced [14, 15] and less experienced, younger [16] German GPs. A key finding of the first study was that experienced GPs used CAM as one of several strategies to deal with “therapeutically indeterminate situations” [14]. Such situations are characterized by two sets of conditions: Firstly, there is a desire for treatment, either by the patient or the physician, or both. Secondly, either such treatment is not (unambiguously) necessary from a medical perspective, or a professionally accepted treatment is not available, or not acceptable to the patient. Typical examples of indeterminate situations are a patient with a (presumably viral) cold requesting antibiotic treatment or a patient’s wish for a treatment for medically unexplained symptom or chronic complaints that have not responded to conventional treatment. All participants in the first study reported that they tried to resolve such situations through empathetic consultations without further treatment, but this strategy was not always sufficient. Some participants used CAM because they were convinced of its effectiveness, while others used it as a non-specific (or placebo) treatment and as a relationship tool (these findings are well in line with those of quantitative studies [6, 7]). Another important but rarely explicitly reported strategy was using conventional treatments (such as antibiotics or pain killers) in a very liberal manner [14]. Important arguments for justifying CAM use were using it as a supplementary tool to conventional medicine, not as an alternative; the experience that evidence and science leave many problems in general practice unanswered; and the wish to help the individual patient, justifying the use of procedures not based on science for therapeutic and communicative purposes; and a strong belief on one’s own clinical experience [15]. The main reason for rejecting CAM modalities was that they were seen as not evidence-based or scientifically implausible. In the second study, it was found that younger GPs (who were still more influenced by medical school and often still struggling with the basic challenges of primary care) tended to be more critical of CAM [15].

### Study aim

In view of the results of these two previous studies [14–16] and the significantly lower use of CAM by GPs in other countries [2–8], we conducted an explorative qualitative follow-up study in which we interviewed GPs who had worked both in Germany and in another European country: either Italy, the Netherlands, Norway or the United Kingdom (UK). Two aims of this study were to investigate (1) what country differences the participants perceived in relation to the role of CAM in general practice and (2) how they explained these differences. The study also looked at perceived country differences in everyday general practice independent of CAM. These findings are reported in detail elsewhere [17], but some central results are included in the following section. In order to illustrate to the reader the extent to which the framework conditions for primary care differ in the five countries, we will first give a brief overview of these differences before moving on to the methods and specific results of our study.

### The framework conditions for primary care in the five countries investigated in the study

All the countries studied are European welfare states, but they differ in their approaches to the organisation and financing of the healthcare system. The healthcare systems in the UK, Italy and Norway are tax-funded and all citizens can automatically claim their benefits. In the Netherlands and Germany funding is provided through compulsory contributions to statutory health insurance [18]. Only in Germany is private health insurance available as an alternative to statutory insurance for people whose annual income exceeds a defined limit. In the Netherlands, Norway and the United Kingdom, GPs have a strong gatekeeper function. This means that specialists usually work at hospitals and are generally only available following a referral from a GP. In Germany, on the other hand, the number of specialists in outpatient care (who are easily accessible within the statutory health care system) exceeds the number of GPs. The Italian healthcare system also provides GPs with an important gatekeeper function [18]. In reality, however, this is considerably weakened by a number of factors (e.g. increasing numbers of outpatient specialists in private practice) [17, 19]. The mechanisms for the payment of GPs are very different in the five countries [17, 19]. In Italy, the majority of doctors’ remuneration is based on per-capita flat rates for registered patients that are independent of performance. In the Netherlands and the United Kingdom, per capita remuneration also accounts for a large proportion of a GP’s income, but it is linked to performance. Furthermore, conformity with guidelines also contributes to income. In Norway, income comes in roughly equal parts from per capita payments from the municipality, fees for services from the central government and co-payments from patients. Fee for service is the main payment mechanism in Germany. Major country differences also exist with regard to practice size and the composition of practice teams [17–19]. In the Netherlands, Norway and the UK, institutes for general practice have existed at all medical faculties for many years. These institutes also have an important influence on post-graduate education. In Germany, such a development has only taken place in the last 20 years. In Italy, on the other hand, not a single medical faculty has an institute of general practice, and the status of general practice within the medical profession and at universities in general is weaker than in the other countries [17, 19].

According to the participants in our study described below, the major differences in the framework conditions and the national peculiarities mean that the day-to-day work of GPs differs greatly from country to country [17]. Several participants described the direct experience of switching countries as “shocking”. In particular, they discussed how the strong differences in remuneration and billing mechanisms set complex incentives that affect their daily profoundly in a variety of ways.

## Methods

### Study design and recruitment

For this explorative qualitative study, we conducted 12 semi-structured individual interviews between September 2018 and August 2019 after the participants had been informed and had given their consent. The study protocol had been approved by the Ethics Committee of the Medical Faculty of the Technical University of Munich (TUM; Project ID 149/18 s). To be eligible, participants had to (1) speak sufficient German for the interview, (2) have completed postgraduate training in general practice/family medicine and (3) have worked as GPs for at least three months in Germany as well as in Norway, UK, the Netherlands or Italy. These countries or regions were selected for two reasons: (1) their health care systems differ significantly from the German system; (2) the authors had good professional contacts, which should facilitate recruitment. Participants should have worked exclusively or most of the time in ‘typical’ primary care practices (first contact and principal point of continuing care in the health care system). Participants were recruited through snowball strategy [20] (for example, some interviewees were identified either with the help of medical colleagues with large professional networks, participating GPs or through internet searches). Additionally, four of the participants were recruited through the professional network of the first author. Qualitative research is always related to the researcher, and self-reflection on one’s own situatedness is essential. The inter-disciplinary study team consisted of a fifth-year medical student (meanwhile medical doctor) with work experience as a qualified nurse (RB), two sociologists (BJ, JG) and one medical doctor/clinical epidemiologist (KL). This means that some of us had actual experiences in medical professional work, while some of us had none. Only KL had extensive experience in (mostly quantitative) CAM research. These different backgrounds and our diverse experiential knowledge, puts us in a unique position that the research team had both insider and outsider perspective on the empirical material. This also meant that we had a varied view of the data material. Further details are reported in the supplementary file according to the Consolidated Criteria for Reporting qualitative Research (COREQ) checklist ​ [21] (see additional file 1).

### Data collection

The interviews were conducted by the second author (RB) either in person (*n* = 3) or by telephone (*n* = 9). A topic guide was used for the interviews (see additional file 2). The participants were first asked to give a brief outline of their medical careers in Germany and the other country. Then they were asked to describe what they considered to be the most important country differences in GP work and primary care in general. In the further course, the participants were asked about country differences regarding the role of CAM in GP, the relevance of evidence-based medicine (EBM) and science, and the handling of indeterminate situations. Interview was designed to be open to participants freely discussing and developing topics that they deemed as significant - this was used intensively. The duration of the interviews was between 33 min and 77 min. The interviews were recorded with a digital audio recorder, transcribed verbatim and pseudonymised.

### Data analysis

The analysis was carried out according to the methods of thematic analysis by Braun and Clarke [22] with the help of the software programme MAXQDA 2020. Thematic analysis focuses on the identification, analysis and description of cross-case patterns or themes in the data that belong together in terms of content. In the first step (“familiarising yourself with your data”), the interviews were read, sometimes several times, by the team members, commented on and ideas for coding were collected. In the second step (“generating initial codes”), an inductively oriented first coding was done primarily by the second author. In the further course (“search for themes”), the codes were assigned to potential themes. After this process was completed, the fourth step (“reviewing themes”) involved an intensive revision and condensation of the theme structure in relation to the research questions, taking both the coded data extracts and the interviews as a whole into account. The individual themes were then further concretised, defined and named (“defining and naming themes”), a process that was only finally completed during the manuscript preparation (“producing the report”). All steps were accompanied by team discussions. In this article, we only address themes that are related to our CAM research questions. For results presentation below, we selected concise quotes. The original quotations were translated into English with due care (KL and JG, also with the aid of DeepL [23]) and are cited with a pseudonymised participant number and the position in the transcript.

## Results

The basic characteristics of the 12 participants in terms of socio-demographics, education and training, migration and medical practice are summarised in Table 1. The patterns of the participants’ characteristics differed between the four countries. For example, all GPs in the Norwegian and British groups had grown up in Germany and all but one had returned to Germany at the time of the interviews. In the Dutch group, all three GPs were born in the Netherlands and had studied medicine there. As participants with experience in Italy, we were only able to recruit doctors from the northern, predominantly German-speaking province of Alto-Adige/South Tyrol (see Box for information). They all were younger than 40 years, had grown up in South Tyrol and had completed their GP specialist training in Germany. The GP practices in which the participants had worked in South Tyrol were all rural single practices, while the locations and size of the practices in the UK, the Netherlands, Norway and Germany were different. As far as could be ascertained, four of the participants reported to be highly skeptical about CAM and/or did not use it in practice, seven held pragmatic views with limited use (at least two of them used it more frequently when working in Germany), and one appeared to be a frequent user who was very open to some CAM methods.

**Table 1:** Characteristics of participants

Participant ID	Gender	Age group	Born in	Medical school in / GP specialist training in	Currently working in	Last country change	Reason for migration	Worked as GP in the other* country for	Attitude towards CAM
NOR-1	Male	41–50 years	GER	GER / NOR	GER	11–20 years	Private	5–10 years	Rather positive
NOR-2	Male	61–70 years	GER	GER / NOR & GER	GER	11–20 years	Private	> 20 years	Skeptikal
NOR-3	Male	51–60 years	GER	GER / NOR & GER	GER	5–10 years	Private	5–10 years	Rather skeptikal
UK-1	Male	51–60 years	GER	GER / UK	UK	11–20 years	Work	< 5 years	Rather positive
UK-2	Male	41–50 years	GER	GER / GER & UK	GER	11–20 years	Private	5–10 years	Rather skeptikal
UK-3	Female	51–60 years	GER	GER/ UK	GER	5–10 years	Private	10–20 years	Rather positive
NL-1	Female	31–40 years	NL	NL / NL	GER	5–10 years	Private	< 5 years	Skeptikal
NL-2	Male	51–60 years	NL	NL / NL	GER	5–10 years	Work	5–10 years	Skeptikal
NL-3	Female	51–60 years	NL	NL / GER	NL	> 20 years	Private	< 5 years	Skeptikal
I-ST-1	Female	31–40 years	I-ST	GER / GER	I-ST	< 5 years	Private	5–10 years	Rather positive
I-ST-2	Male	31–40 years	I-ST	Austria / GER	GER	5–10 years	Work	< 1 year	Positive
I-ST-3	Male	31–40 years	I-ST	Unklar / GER	I-ST	< 5 years	Private	5–10 years	Rather positive

GER = Germany; NOR = Norway; UK = United Kingdom; NL = Netherlands; I-ST = Italy – Southern Tirol/Alto Adige

* not the country where work is currently being done (at the time of the interviews)

**Box:** Basic information about South Tyrol

South Tyrol (Italian: Alto Adige) is an autonomous province in the north of Italy with approximately 530,000 inhabitants. For historical reasons, there are three official languages. According to official statistics [24], 69% of the population belong to the German, 26% to the Italian, and 5% to the Ladin language group.
The province is located in the middle of the Alps and more than half of the population lives in rural and mountainous areas. Central sources of income are tourism, agriculture and services. The basic health system of South Tyrol is the same as all over Italy.

In the following, we will first present how participants compared the perceived relevance of CAM for general practice in Germany and the other countries – our “context theme” that we need to interpret all other themes. Then we discuss the four main themes summarizing the perceived reasons for the country differences (see Table 2 for an overview of the themes per country). Germany usually represents the reference point against which the respondents described their experiences. At the end of the results section, we briefly summarise the few statements in which participants explicitly expressed their personal views on CAM in relation to country differences.

**Table 2:** Overview of the themes and how they were expressed for the individual countries

	Italy/South Tyrol	Netherlands	Norway	UK	Germany
**Context theme**					
The relevance of CAM for general practice	In Italy less than in Germany, but South Tyrol special	Less than in Germany	Less than in Germany	Less than in Germany	Reference contrast
**Main themes: perceived reasons for coutry differences**					
Importance of science, EBM and guidelines in relation to CAM	Less than in Germany – not discussed in relation to CAM	Stronger than in Germany – strong counterpart	Stronger than in Germany – strong counterpart	Stronger than in Germany – strong counterpart	Reference contrast
Patient-provider communication to resolve indeterminate situations	Not discussed in relation to CAM	Trained and utilised more efficiently than in Germany – reducing need for CAM	Trained and utilised more efficiently than in Germany – reducing need for CAM	Trained and utilised more efficiently than in Germany – reducing need for CAM	Reference contrast
The influence of patient expectations of GP care	Different expectations of German- and Italian-speaking patients	CAM not seen as the task of GPs	CAM not seen as the task of GPs	CAM not seen as the task of GPs	Higher demands on the healthcare system in general / CAM more expected from GPs
CAM-relevant country differences between health systems	Reference contrast to Germany /no CAM reimbursement	Reference contrast to Germany / payment of time	Reference contrast to Germany / More time per patient	Reference contrast to Germany /no CAM reimbursement	Stronger entrepreneurial focus / some CAM modlities reimbursed
**Relevance on the personal level**					
Perceived deficiencies when CAM is not an option	Adressed	Adressed	Not adressed	Adressed	Reference contrast

Reference contrast means that the topic was explicitly described for the other country, while the reference country was primarily characterised indirectly via the contrast

### Context theme: the relevance of CAM for general practice

Our context theme arose directly from the participants’ answers to the question in the interview guide on country differences in the role of CAM in general practice. The relevance (we use this term because we consider it narrower than “role”) of CAM in general practice was mostly described in terms of how many or how often doctors in a country use CAM compared to Germany, and whether CAM was considered an important part of daily work. In line with the wording of our key question, participants were primarily referring to GPs in the country and their opinion of CAM in general, rather than their own attitudes and use.

Unanimously, participants with work experience in the Netherlands, Norway and UK reported that CAM had less relevance in general practice in these countries compared to Germany. For example, one of the GPs with professional experience in Norway pointed out that “*[CAM] is used much less than in Germany” (NOR-1*,* pos. 4).* Similarly, an interviewee who worked in the Netherlands emphasised the low number of GPs using CAM: *“There are [Dutch] GPs who use CAM*,* but probably very few” (NL-3*,* pos. 42).* A GP still working in the UK said *“unlike in Germany*,* there is no culture of using complementary procedures” (UK-1*,* pos. 76).* Acupuncture was the only CAM treatment method that was considered to be of some importance for general practice in the interviews in Norway and the UK (no such statements were made for the Netherlands).

As in the Netherlands, Norway and UK, the relevance of CAM in Italian general practice and its role in Italy in general were considered smaller than in Germany.

One participant reported that CAM is not nearly as widespread [in Italy] as in Germany (I-ST-1, Pos. 90).

However, cultural and geographical factors had an important impact. In South Tyrol, CAM actually had some relevance in the work among German-speaking patients. This will be described below in the section “The influence of patient expectations of GP care”.

In summary, all participants agreed that the relevance of CAM for GP practice is lower in the other four countries compared to Germany. This context is important for the following four themes, which cover the perceived reasons for differences between countries in relation to CAM.

### Importance of science, EBM and guidelines and its relation to CAM

Country differences with regard to the importance of science, evidence-based medicine and guideline compliance for daily medical practice were a central theme in the interviews comparing Germany with the Netherlands, Norway and the UK, both in terms of GP work in general and as a factor influencing the relevance of CAM.

The participants reported that in these countries science, and more specifically EBM and EBM-based guidelines, are considered more important by GPs and the healthcare system than in Germany. At the same time, CAM was seen as not evidence-based and not scientific. The participants mostly spoke of CAM in general. Only in exceptional cases a distinction was made between individual CAM modalities. One of the participants with experience in Norway pointed out the orientation towards the Anglo-Saxon medical culture:

I think that the Norwegian health system is more Anglo-Saxon, it is more oriented towards Great Britain and the USA, and in this respect is also more faithful to science, saying: “We only want to practice a medicine that has been scientifically tested”. In this respect, homeopathy and natural medicine have a very difficult time in Norway. (NOR-2, pos. 66)

One of the participants with work experience in the Netherlands claimed in a very general way that there is no evidence that patients really benefit from CAM:

The most important reason [that CAM is almost never used by Dutch GPs]: It has not been scientifically proven that it is good for patients (NL-2, pos. 80)

Not all participants had this strong disapproval of CAM in general and described it more neutrally as a characteristic of a country’s medical culture and healthcare system. However, for the Netherlands, Norway and the UK, the view that CAM is not considered evidence-based, almost by definition, was seen as a normal attitude that needs no further explanation or discussion. None of the participants reported having read studies on CAM themselves.

Following guidelines was considered as implementing science and EBM into clinical practice. And following the guidelines meant not using CAM (under the implicit assumption that guidelines do not recommend CAM modalities).

So somehow I have the feeling that here [in Germany] you have science on the one hand and general practitioners on the other, and there’s a huge gap in between. I would like to stand in this gap, I think both are great and I always want to combine them in this way, and in the Netherlands this was somehow taken for granted and yes, as I said, the guidelines are read, they are learned and followed. (NL-1, pos. 67)

EBM and guidelines were considered increasingly important in Germany, too. In the other three countries, however, EBM was perceived to be more effective in connection with the stronger gatekeeping role of GPs, the greater emphasis on the economical use of limited resources and a more utilitarian perspective. Germany was described as a country that gives individual doctors more opportunities to do what they consider appropriate for the individual patient, including the use of CAM. One participant spoke of a “romantic medical tradition”:

That’s a huge difference [between Germany and the UK]. In Germany, there is a romantic medical tradition that has run through all kinds of systems… Holism and so on. And in Britain it’s very, very pragmatic, very bureaucratic and very scientific. … everything goes very, very hard according to evidence-based medicine. (UK-1, pos. 38)

The participants from Italy/South Tyrol, on the other hand, stated that EBM and guidelines play a greater role for GPs in Germany than in their home country. But these statements always referred to general practice in general. No connection was ever made with CAM use.

In South Tyrol evidence-based medicine is less established than in Germany. (I-ST 3, pos. 49).

In summary, participants with work experience in the Netherlands, Norway and the UK considered the strong EBM and science orientation in these countries as the most important reason for the low use of CAM by GPs compared to Germany. In Italy/South Tyrol the relevance of EBM was perceived as low, but this was not discussed as a factor influencing CAM use by Italian GPs.

### Patient-provider communication to resolve indeterminate situations

A second important explanation of the participants with working experience in the Netherlands, Norway and UK as to why CAM is less used (or needed) by GPs in these countries compared to Germany was the role of the patient-provider communication in resolving indeterminate situations.

All three countries have well-structured GP specialist training programs that are both scientifically and practically oriented. These were highly praised, particularly in the interviews with the Netherlands and the United Kingdom, and were seen as significantly better than the training programmes available in Germany at the time. Patient-provider communication had an important role there and was regularly trained. For example, a participant working in the UK said:

You have video recordings of your consultations, you have a trainer who deals with you personally, so this extent of reflective medicine and personal medicine, in which relationships with patients have a value, that was almost like an enlightenment for me. (UK-1, pos. 12)

According to the participants, this training had helped them to better (than German GPs) explore and understand better what patients expect from the GP, to reassure them and to avoid unnecessary diagnostics and/or treatment, whether conventional, complementary or alternative. This was particularly important to the participants when answering the question about country-specific differences in dealing with indeterminate situations (the interviewer had explained this term to the participants using examples).

How do I deal with “indeterminate” illnesses is a constant topic in Norwegian GP specialist training. And it is also clearly stated there: “So it is important that you can do this without medicalising a condition, i.e. without attaching a medical label to it too quickly.” So [the GP] is also much less likely to say: “This is an anxiety disorder, or this is a phobia, or this is fibromyalgia.” (NOR 2, pos. 48)

In the Netherlands, communication is the “be-all and end-all”. During postgraduate education, one day a week you go to university still and half a day of that is communication lessons, for three years. You can’t imagine that here [in Germany], it’s simply a completely different effort. And what’s always so important: “Why is this patient coming to me now?” They often just want this sick note and reassurance that it’s not pneumonia but that it’s simply a cold and that they have to wait it out. But here physicians always immediately reach for the prescription pad and prescribe something, because I have to do something and this patient is sitting here across from me now. (NL-1, pos. 50)

So, in the perception of participants with work experience in the Netherlands, Norway and the United Kingdom, attitudes and communication strategies acquired during specialist training contributed significantly to the fact that CAM modalities (and unnecessary conventional measures) were used less frequently in these countries than in Germany. In the interviews on Italy, a connection between patient provider communication and CAM use was not mentioned by the participants.

### The influence of patient expectations of GP care

Although our interview guide focussed on the GP perspective, most participants also discussed aspects related to the role of patients in the differences between countries. Regarding patient expectations of GP care, three subthemes were addressed in the interviews: the level of expectation in general, whether CAM is expected from the GP and, in the case of Italy/South Tyrol as a very specific subtheme, differences between the language groups.

In general, several participants stated that expectations of the healthcare system were higher in Germany than in Norway, the Netherlands and the UK. Specifically in relation to GP care, interviewees reported that their experience was that patients in Germany often seek treatment for very minor complaints, expected a quick referral to specialists and have high expectations of receiving treatment. From the participants’ point of view, this created pressure to act, opening the door to measures that “do not stand up to scientific scrutiny” but fulfil patients’ expectations.

… the pressure to leave the practice with a prescription or anything else tangible is also much less [in Norway] than in Germany. … the temptation to offer something tangible, but which hardly stands up to scientific scrutiny, I have experienced this much more in Germany. (NOR-2, Pos. 54)

Respondents did not explicitly report a particularly strong active demand for CAM by German patients. Instead, they often said that there was interest in CAM procedures from patients in the other countries as well. But patients in these countries did not expect to receive CAM from their GP. And GPs also did not see it as their job to offer CAM. Instead, it was normal for patients to seek such treatments elsewhere, e.g. from doctors specialising in CAM or from non-medical providers. German and South Tyrolean GPs, on the other hand, seemed to regard this more often as part of their work.

But [in the UK] the patients who wanted to be treated homeopathically actually went to an appropriate doctor (UK-2, pos. 22)

This “complementary” part, the GPs here [in Germany] feel responsible for that, and that’s not the case in Holland, … that’s “not my cup of tea.” (NL-1, pos. 93)

In the interviews with South Tyrolean GPs the main theme regarding patient expectations were differences between the language groups. According to the participants, Italian-speaking patients often rated their symptoms as more severe than German-speaking patients and were more likely to demand strong conventional treatment approaches. Italian-speaking patients were also described as less open to CAM than German-speaking patients, who have often already had used and actively asked the GP for CAM or traditional treatments (the term “home remedies” was used in all three interviews). Home remedies were described as widespread, especially in German-speaking remote rural areas. As a consequence, in a subgroup of patients CAM actually has considerable importance in South Tyrol.

I also find in practice that Italian-speaking patients … often assess the symptoms as more serious than German people or patients, so to speak, and at the same time had higher expectations of therapy or medication or antibiotics than German patients here now. (I-ST 2, pos. 32)

Italians are not as open to [CAM] approaches as German speakers. So I often hear from German speakers: “Yes, aren’t there any [homeopathic] globules for that?” or they already have some globules at home and have used them and I’ve never heard that from Italians. (I-ST-1, pos. 90)

Overall, from the participants’ perspective, patients’ expectations can have an important influence on the relevance of CAM in GP practices. However, this appeared to be part of a complex web of cultural and health system-related framework conditions that have a general impact on GP care in a country.

### CAM-relevant country differences between health systems

Differences in health systems and the related framework conditions for general practice were also discussed as factors influencing CAM use and the handling of indeterminate situations. Due to regulatory conditions and remuneration mechanisms, German GP practices were perceived as being more entrepreneurial, independent small businesses than GP practices in the other four countries. One participant referred to this as the “shopkeeper model” (UK-1, pos. 70). Fee-for-service remuneration was described as a central part of the income of German GPs. Some CAM treatments are covered by social health insurance (e.g. acupuncture for chronic low back pain). Other CAM modalities and conventional diagnostic or therapeutic measures not covered by social health insurance can be offered as “IGEL services” (abbreviation of the German term “Individuelle Gesundheits-Leistungen” = individual health services) which have to be paid by patients out of pocket. In the view of some participants, this increases the interest of GPs in learning and applying CAM modalities. Such a “culture” does not exist in the other four countries or only to a smaller extent.

Unlike in Germany, there is no culture [in the UK] in which complementary measures and IGEL services are then [in indeterminate situations] used. (UK-1, pos. 76)

Participants from Norway and the Netherlands discussed another “system condition” which is relevant to the handling of indeterminate situations: the remuneration of consultation time. In their view, having enough time for a patient reduces the need to use CAM modalities or unnecessary conventional interventions. In the Netherlands, it is possible to charge longer consultation times if required. Norwegian patients have to pay a fee for each GP visit. However, as one of the participants put it, this also buys them the usual 20-minute time slot.

Whoever pays is always entitled … a time slot of at least twenty minutes. (NOR-3, pos. 32)

In Germany, GPs have significantly less time per patient, which the participants believe leads to more CAM being prescribed.

### Relevance on the personal level - perceived deficiencies when CAM is not an option

When talking about the country differences participants mostly referred to the prevailing views in the respective countries. On a personal level the majority felt CAM modalities can be a helpful tool for general practice, but only a few interviews explicitly addressed personal views in relation to country differences. In these cases, there was some regret when CAM was not available as an option. Two of the South Tyrolean participants regretted, for example, that herbal medicines are less readily available in Italy than in Germany. On a more specific level, two participants discussed that, if CAM is not available as an option, some patients with high and lasting symptom burden for whom an accepted treatment is not available are left alone by their GPs. One of the participants - a very sceptical Dutch GP who does not use CAM - noted this with some regret.

To be honest, in the Netherlands we leave such patients relatively alone and say: “We just don’t know anything about that, I don’t know any alternative medicine.” (NL-1, pos. 87)

A participant from the UK liked to use CAM procedures such as manual therapy or acupuncture with such patients in order to address the patient’s suffering on a different physical and communicative level.

[When using manual treatment or acupuncture] I interact with the body under the premise that I am doing something in the here and now to reduce the level of suffering. I’m in a completely different communication situation with the patient. …. And that’s very, very unusual here [in the UK]. Most of the procedures here are about giving pills and not touching. (UK-1, Pos. 88 & 96)

## Discussion

### Summary of main findings

Participants unanimously reported that they perceived CAM to be more relevant in general practice in Germany compared to the other countries. We identified four overarching themes in relation to the perceived reasons for these differences. Firstly, physicians with experiences in countries with a strong EBM and science orientation (Netherlands, Norway and the UK) considered the deeply ingrained view in national healthcare systems and GP communities that CAM modalities are not evidence-based as the main reason for the lower use of CAM by GPs. Secondly, extensive training of communication skills was cited as a reason that reduced the need for CAM in the Netherlands, Norway and the UK. Thirdly, differences in patient expectations and demands were perceived as a factor contributing to greater utilisation of CAM by German GPs compared to the other four countries. Finally, country-specific reimbursement mechanisms were considered as a factor influencing the role of CAM in general practice.

### Strengths and limitations

The main strength of our study is that participants had practical experience as GPs in both of the countries they compared. We are not aware of any studies in general practice with a similar approach. However, this strength goes along with several important limitations. The number of interviews per country was small, making theoretical saturation unlikely when themes became country-specific. At which phase in their career, how long and in which sequence participants worked in Germany or the comparator country was highly variable, and the pattern of characteristics varied between the countries. For Italy, we were only able to identify eligible participants in a particular province, which in many ways is not representative of the country as a whole. For our CAM question, the Italian interviews yielded relatively little material (in contrast to the general country differences in primary care). When doing the first interviews we were overwhelmed and not really prepared for the detailed description of very large country differences in relation to working as a GP in general. This also meant that our initial CAM focus was rather secondary to the overall country differences for the participants. But we accepted this shift in focus because the authentic accounts of the participants seemed very important to us. Our study only investigated the perspective of GPs. The patient’s perspective is missing, as well as a historical and sociological perspective that focuses on the emergence and social contexts of the different “medical cultures”. Finally, our study covers only five European countries. These limitations mean that our results can only be considered as a first indication of how health systems, primary care settings and socio-cultural factors might be related to the use of CAM in a country.

### Interpretation

Comparing the use of CAM modalities both among GPs and populations in different countries in a valid manner is difficult for various reasons. International comparative studies are either rare (for the general population; e.g. [1]) or non-existent (among GPs). The definition of CAM is vague in general [25] and variable between countries even within Europe [26]. As a consequence, attempts to develop internationally usable instruments for quantifying CAM use [27] have been discussed critically [28]. Therefore, the comparison of CAM use among GPs in the five countries addressed in our study has to rely on national data of variable quality and actuality. National surveys among randomly sampled GPs are available for Germany and England [2, 6, 7], and a regional survey for the Italian region Tuscany [5]. For Norway a recent survey in a random sample of the general population inquired whether participants received CAM from physicians [29]. For the Netherlands we could only identify studies which addressed other aspects of CAM in primary care which give indirect hints on the (low) CAM use by Dutch GPs (for example, [30]). But even when taking all the problems of the scarce data into account the available evidence quite clearly suggests that – as reported by our study participants – the relevance of CAM in general practice in Germany is much higher than in the other four countries.

A remarkable finding of our interviews was the very consistent narrative with regard to the Netherlands, Norway and the United Kingdom that a stronger scientific and EBM orientation is seen as the main reason for the lower utilisation of CAM by GPs compared to Germany. While we could not find any systematic comparative analysis of the importance of EBM across countries, the available national health system reviews of the five countries addressed in our study indeed suggest that EBM is more systematically implemented in health policy-making and health systems in the Netherlands, Norway and UK than in Germany and Italy [31–35]. In the Netherlands, Norway and UK, university institutes for general practice have long been established at all medical faculties [19] and often have a high research output [36]. Together with the strong gatekeeping-keeping function of GPs this gives the discipline a stronger position within the medical profession than in Germany and Italy [19]. For example, an article on the Dutch health care system describes general practice as “the leading force in EBM” in the Netherlands [37]. Quantitative and qualitative studies among GPs have reported an association between a perceived lack of evidence and plausibility of CAM and not using CAM (e.g. [7, 38]). Our findings suggest that in the Netherlands, Norway and UK the view that there is no evidence for CAM is strong and shared by many GPs. Interestingly, our study participants did not discuss the evidence base for or against specific CAM modalities. This aspect also appears relevant as, although there is limited evidence for CAM in many areas, it is not uncommon for high-quality guidelines to recommend specific CAM interventions based on the findings of clinical trials (e.g. [39]). Also, the considerable weaknesses of the evidence base of many conventional treatments [40] were not discussed. This suggests that what is considered evidence- or science-based is influenced less by a careful assessment of the actual data than by mainstream opinion in the respective countries.

In our earlier study of experienced German GPs [11, 12], the majority of participants had reported that a major reason for using CAM was that they were not well prepared for the frequent indeterminate situations encountered in practice. They interpreted this experience as evidence of the limitations of the biomedical approach and became very pragmatic and open to unorthodox problem solving. During the time in which these doctors were being trained, GP training in Germany was much less structured and science-orientated than in the UK, the Netherlands or Norway. In our current study, study participants with work experience in these three countries reported how communication skills trained during GP specialist training reduced their need to use CAM. This fits very well with the position of many academic representatives of the discipline that understanding the consultation from the patient’s perspective and shared decision-making are of central importance for dealing with uncertainty and ambiguity in general practice [41]. The reports of our study participants on communication were often embedded in a praise of the GP specialist training programs. It generally seemed that these programs played an important role in strengthening and stabilizing a professional self-image in which science and EBM orientation, adherence to guidelines and scepticism with unconventional means have an important role.

Interestingly, our South-Tyrolian participants reported that guidelines and evidence-based medicine are less important in general practice in Italy than in Germany. In Italy, there are no institutes of general practice at universities, and since only universities can accredit specialists in this country, there is no specialist designation for general practice. Postgraduate training is organized by the regions and regional associations of GPs [19]. Therefore, the main reason for the country difference between the Netherlands, Norway and UK in comparison to Germany did not apply for Italy.

As our study focussed on the GP’s perspective, our material provides only limited information on the influences of attitudes and expectations of patients or national populations, and of the national cultural, legal, political and historical context. It is very likely that factors related to these issues have a major influence on CAM use in a country [1]. They are also likely to have an important impact on GPs and how they experience country differences. The discussions of our South Tyrolean participants about the different role of CAM in German and Italian-speaking patients provides a small insight into this complex field. What our study results suggest is that the demand for CAM in GP practices in Germany is probably greater than in the other four countries.

The reports from our participants point to very large differences between countries in terms of the general framework conditions for primary care [17]. However, only a few individual regulatory details were explicitly discussed in the interviews in relation to CAM. The comparatively strong entrepreneurial orientation of German general practices and the billability of at least some CAM treatments were seen as favourable for the use of CAM. It is likely that a number of other system factors (and their interplay) also have an important influence but were not discussed in the interviews.

We consider our findings to be original and interesting, but not least because of the methodological weaknesses of our study, they raise more questions than they answer. The approach of interviewing people who have actual experience in two or more countries seems very promising to us. This allows country differences to be perceived on a completely different level than in formal system comparisons or separate studies in individual countries. However, in order to carry out such studies more systematically and on a larger scale, international co-operations and adequate financial resources are essential. Such studies could then not only help to better understand the differences between countries, but also to identify starting points for changes to the framework conditions.

## Conclusion

The study results point to major differences between countries with regard to the role of CAM in GP care. Differences in basic attitudes in the discipline of general practice, patient expectations and system conditions appear to play an important role here. Larger studies with both lay people and GPs who have lived in different countries could provide deeper insights.

## Electronic supplementary material

Below is the link to the electronic supplementary material.

Supplementary Material 1

Supplementary Material 2

## Data Availability

The full dataset is available from the corresponding author on reasonable request.
