# Supplementary material for: How does the role of complementary and alternative medicine in general practice differ between countries? Interviews with doctors who have worked both in Germany and elsewhere in Europe
Source: BMC Complement Med Ther. 2024 Sep 3;24:328. doi: 10.1186/s12906-024-04624-w (PMC11373194; doi:10.1186/s12906-024-04624-w)
Supplement: Supplementary file 1 — Supplementary Material 1 [file 12906_2024_4624_MOESM1_ESM.docx]

**COREQ Reporting Checklist**

| Domain 1: Research team and reflexivity | |
| --- | --- |
| Personal Characteristics | |
| 1. Interviewer/facilitator | Robert Bayer; see Methods Section “Data Collection”. |
| 1. Credentials | RB: cand. med. at the time of interviews, now MD  BJ: Master of Arts Sociology, Dr. rer. soc.  JG: Master of Arts Sociology  KL: Prof. Dr. med. |
| 1. Occupation | RB: medical student at the time of the study, part time work as nurse in a psychiatry ward; now physician  BJ, JG: research associates  KL: Research coordinator |
| 1. Gender | RB, JG, KL: Male  BJ: Female |
| 1. Experience and training | RB: had previous training and professional experience as nurse, studied medicine. As interviewer he was trained by an experienced qualitative researcher and by a medical student having done GP interviews in a previous study. Participated in qualitative workshops.  BJ: University degree and Dr. rer. soc. in sociology; significant experience in qualitative research and interviews  JG: University degree in Sociology; significant experience in qualitative research and interviews  KL: substantial experience in quantitative and qualitative research; conceptualization and lead of several projects |
| Relationship with participants | |
| 1. Relationship established | Four interviews were known prior to KL and contacted by him. The other authors (RB, BJ, JG) had no prior existing relationship with the interviewees. |
| 1. Participant knowledge of the interviewer | Interviewees were informed about the project, interviewer’s educational background and occupational status in advance. |
| 1. Interviewer Characteristics | See 3 & 5 |
| Domain 2: Study design | |
| Theoretical Framework | |
| 1. Methodological orientation and theory | Reported in the methods section “Data analysis”:  The transcribed interviews were analysed using thematic analysis according to Braun & Clarke. |
| Participant selection | |
| 1. Sampling | Reported in the methods section. |
| 1. Method of approach | Reported in the methods section. |
| 1. Sample size | Reported in the methods section. |
| 1. Non-participation | One potentially eligible doctor, who had initially responded positively to an initial contact, did not respond to the repeated formal invitation to participate (reason unclear) |
| Setting | |
| 1. Setting of data collection | Reported in the methods section “Data collection”:  The interviews were conducted either in person (n =3) or by telephone (n=9). |
| 1. Presence of non-participants | No one else was present besides the participants and the interviewer. |
| 1. Description of Sample | See Table 1. |
| Data Collection | |
| 1. Interview Guideline | See Supplement 2. |
| 1. Repeat interviews | No repeat interviews were conducted. |
| 1. Audio/Visual recording | Reported in the methods section “Data collection”:  Interviews were audio-recorded and transcribed verbatim, including pseudonymization. |
| 1. Field notes | Only basic field notes were written. |
| 1. Duration | Reported in the methods section “Data collection”:  Interviews lasted between 33 and 77 minutes. |
| 1. Data saturation | Data Saturation is discussed in the section “Strengths and limitations”. |
| 1. Transcripts returned | Transcripts were not returned. |
| Domain 3: Analysis and findings | |
| Data analysis | |
| 1. Number of data coders | Indexing interviews and analysing: RB, JG, KL  Summarizing and charting of the indexed data: RB, BJ, JG, KL |
| 1. Description of the coding tree | Not reported. Figure 1 gives an overview of the dominant themes. |
| 1. Derivation of themes | Reported in the methods section “Data analysis”. |
| 1. Software | Reported in the methods section “Data analysis”. |
| 1. Participant checking | Not reported. |
| Reporting | |
| 1. Quotations presented | Quotations from different participants are presented to illustrate the findings, and are cited with a pseudonymised participant number and the position in the transcript. |
| 1. Data and findings consistent | Yes. |
| 1. Clarity of major themes | Reported in the methods section “Data Collection”:  Most important differences in GP work in primary care; Differences regarding CAM in GP; Relevance of evidence-based medicine and science; Dealing with indeterminate situations in GP work. |
| 1. Clarity of minor themes | As far as the word count permits, we discuss minor themes too. |
