# Supplementary material for: How does the role of complementary and alternative medicine in general practice differ between countries? Interviews with doctors who have worked both in Germany and elsewhere in Europe
Source: BMC Complement Med Ther. 2024 Sep 3;24:328. doi: 10.1186/s12906-024-04624-w (PMC11373194; doi:10.1186/s12906-024-04624-w)
Supplement: Supplementary file 2 — Supplementary Material 2 [file 12906_2024_4624_MOESM2_ESM.docx]

**Interview guide**

**Topic 1 (introductory block): Brief report on GP work/experience in the respective country and in Germany**

Key question: Can you report a bit about your medical career and work in x and D?

**Topic 2: The most important differences between the countries in terms of GP practice and primary care**

Key questions: In your opinion, what are the most important differences between x and Germany when it comes to working as a GP or in primary care?

**Topic 3: Indeterminate situations/responsibility**

Key questions: How do you (and other GPs) in x deal with indeterminate situations (see below for examples if applicable)? Do German doctors feel more/less "responsible" than doctors in x?

**Topic 4: CAM**

Key questions: What role does CAM play in GP care in X? How do you see it in contrast to Germany?

**Topic 5: Science orientation and system differences**

Key question: How do you assess the role of evidence-based medicine and science in X in relation to Germany? What (other) system attributes characterise the differences?
